# Methodological Approaches to Evaluate Teratogenic Risk Using Birth Defect Registries: Advantages and Disadvantages

**DOI:** 10.1371/journal.pone.0046626

**Published:** 2012-10-03

**Authors:** Fernando A. Poletta, Jorge S. López Camelo, Juan A. Gili, Emmanuele Leoncini, Eduardo E. Castilla, Pierpaolo Mastroiacovo

**Affiliations:** 1 ECLAMC (Estudio Colaborativo Latinoamericano de Malformaciones Congénitas) at Centro de Educación Médica e Investigaciones Clínicas (CEMIC) (CONICET), Buenos Aires, Argentina; 2 ECLAMC at Instituto Multidisciplinario de Biología Celular (IMBICE) (CIC-CONICET), La Plata, Argentina; 3 Headquarters of the International Clearinghouse for Birth Defects Surveillance and Research, Rome, Italy; 4 ECLAMC at Instituto Oswaldo Cruz, Rio de Janeiro, Brazil; 5 INAGEMP (Instituto Nacional de Genética Médica Populacional), Rio de Janeiro, Brazil; University of Dayton, United States of America

## Abstract

**Background:**

Different approaches have been used in case-control studies to estimate maternal exposure to medications and the risk of birth defects. However, the performance of these approaches and how they affect the odds ratio (OR) estimates have not been evaluated using birth-defect surveillance programmes. The aim of this study was to evaluate the scope and limitations of three case-control approaches to assess the teratogenic risk of birth defects in mothers exposed to antiepileptic medications, insulin, or acetaminophen.

**Methodology/Principal Findings:**

We studied 110,814 non-malformed newborns and 58,514 live newborns with birth defects registered by the Latin American Collaborative Study of Congenital Anomalies (ECLAMC) between 1967 and 2008. Four controls were randomly selected for each case in the same hospital and period, and three different control groups were used: non-malformed newborns (HEALTHY), malformed newborns (SICK), and a subgroup of SICK, only-exposed cases (OECA). Associations were evaluated using OR and Pearson's chi-square (P<0.01). There were no concordance correlations between the HEALTHY and OECA designs, and the average OR differences ranged from 3.0 to 11.5 for the three evaluated medicines. The overestimations observed for HEALTHY design were increased as higher OR values were given, with a high and statistically significant correlation between the difference and the mean. On the contrary, the concordance correlations obtained between the SICK and OECA designs were quite good, with no significant differences in the average risks.

**Conclusions:**

The HEALTHY design estimates the true population OR, but shows a high rate of false-positive results presumably caused by differential misclassification bias. This bias decreases with the increase of the proportion of exposed controls. SICK and OECA odds ratios cannot be considered a direct estimate of the true population OR except under certain conditions. However, the SICK and OECA designs could provide practical information to generate hypotheses about potential teratogens.

## Introduction

Different case-control study designs have been used to estimate the risk of birth defects after medication exposure. These approaches differ in their definition and selection of the control group. Case-control studies with healthy newborn controls are widely used, especially for studying rare events such as birth defects. In South America, the Latin American Collaborative Study of Congenital Anomalies (ECLAMC) has maintained a surveillance programme for birth defects since 1967 using a case-control design [1].

However, as has been laid out in previous works [2]–[7], these risk estimates from case-control studies are vulnerable to selection bias, confounding bias, and information bias (differential misclassification bias). In this sense, recall and interviewer bias (two types of information bias) are subjects of great concern in birth-defect epidemiology [8]. Recall bias may occur when mothers of babies with birth defects carefully report the use of medications or when they are thoroughly interviewed regarding medicine use as a possible cause of their infants' defects. In the latter case, mothers are more likely to recall medication exposure than are mothers of healthy controls with similar medication use [9]. Interviewer bias arises when the interviewers know who are the mothers of cases and who are the mothers of controls, and then the interviewers may have a higher tendency to determine the exposure histories of cases than the exposure histories for controls. Both biases may result in the over-estimation of the effect of medication (odds ratio) and a higher probability of false-positive results. Although previous studies have found little evidence of differential misclassification of exposures in case-control studies of birth defects (see refferences in Swan et al. [10]), potential reporting bias is a reasonable issue to be considered in studies to assess teratogenic effects of medications.

A useful system have been proposed to post-marketing surveillance of fetal effects of medications using available sources from existing birth defect surveillance programs and globally organized through the International Clearinghouse for Birth Defects Surveillance and Research (ICBDSR) [11]. Considering that methodology, coverage, and sources of ascertainment vary among these birth defects surveillance programs, approaches based on malformed controls and only-exposed controls has been suggested as practical designs to disclose potential teratogens [11]–[14].

The interpretation and usefulness of the epidemiological methods that include healthy and malformed controls have been discussed previously [11], [15]–[18]; however, the performance of these approaches and how they affect the odds ratio estimates have not been evaluated using birth-defect surveillance programmes.

The aim of this study was to evaluate, using the ECLAMC's surveillance programme, the scope and limitations of three approaches to assess the teratogenic risk for birth defects in mothers exposed to specific medications during the first trimester of pregnancy: (a) antiepileptics, which are medications associated with a risk of birth defects; (b) insulin, which is a marker for pre-gestational diabetes, a chronic condition that is well known to be associated with birth defects; and (c) acetaminophen, which is a medication that is not associated with birth defects.

## Materials and Methods

### Sample and case definition

Live-birth cases were those that were registered by the ECLAMC network, involving 102 maternity hospitals from 11 South American countries from 1967 to 2008 and covering 3,939,474 births. A total of 58,514 live births with isolated or multiple birth defects were registered with ICD-X BPA codes [19]. Non-isolated (multiple malformed) cases were counted separately for each type of birth defect. Cases with aetiologic syndromes [20] and those with only a minor birth defect were excluded.

A total of 110,814 non-malformed newborns from the same database were used as healthy controls. Data regarding medication use and illnesses during pregnancy were obtained by qualified physicians using standard interviews of the mothers before their discharge from the hospital at which they had given birth. Data were collected, reviewed and coded by the ECLAMC following the same standardized procedure used since 1967 [1]. Medicines were coded with the standard ATC system [21]. The study protocol was approved by the ethics committee at CEMIC (*DHHS*-IRB #1745, IORG #1315).

### Medicine exposure

The three medicines analyzed in this study were the following: antiepileptics (ATC code: N03A), including valproic acid (N03AG01); insulin (ATC code: A10A); and acetaminophen (ATC code: N02BE01). Exposures to vitamins and iron were excluded from the analysis in order to minimize bias, as exposure to these medications has not been proven to be teratogenic.

### Epidemiological Designs

**Figure 1:**
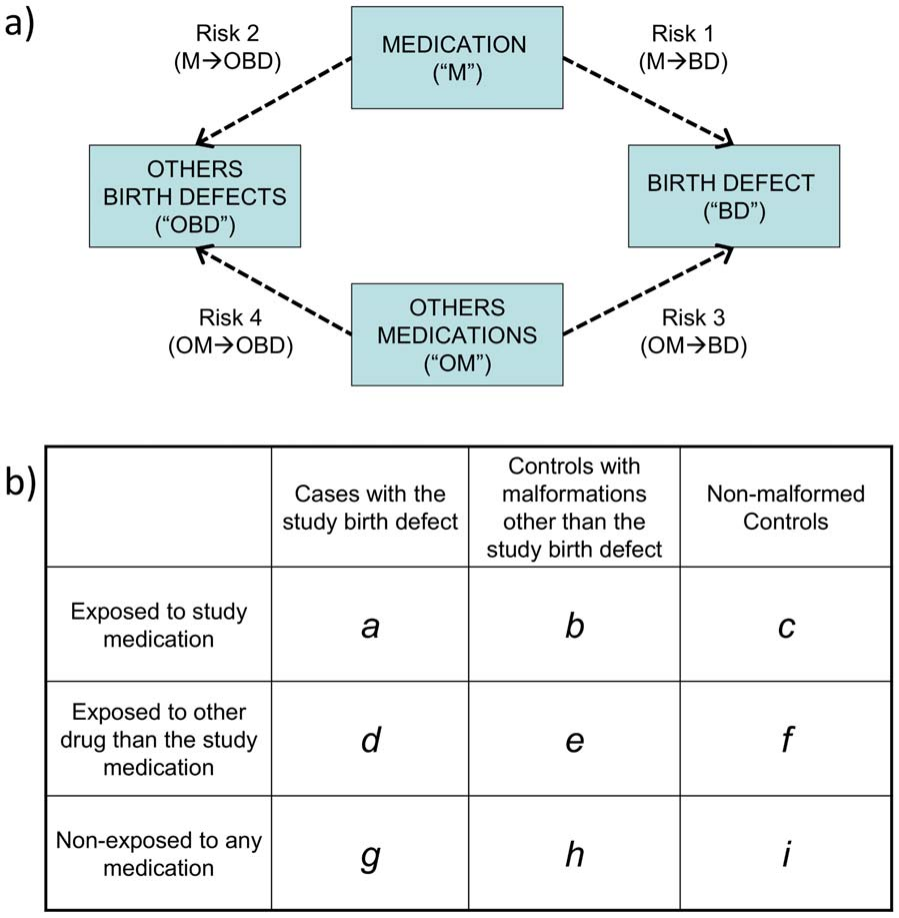
Prenatal exposure to medications and birth defects occurrence. (**a**) Potential relationships between prenatal exposure to medications and birth defects occurrence in the population; (**b**) Three-by-three contingency table of malformed and non-malformed newborns with prenatal exposure to the study medication, exposure to other medications, and non-exposed; cell frequencies are represented by the letters *a*, *b*, *c*, *d*, *e*, *f*, *g*, *h*, and *i*.

Figure 1a shows the four categories of association between the medications and types of birth defects in the sample. Risk 1 (M→BD) is the risk that the study medication (“M”) causes the birth defect studied (“BD”); Risk 2 (M→OBD) is the risk that the study medication (“M”) produces congenital anomalies other than the birth defect under study (“OBD”); Risk 3 (OM→BD) is the risk that other medicines (“OM”) produce the birth defect studied (“BD”); and Risk 4 (OM→OBD) is the risk that other medications (“OM”) cause other birth defects (“OBD”). In prospective studies, these associations could be estimated from the relative risks (*RR*). Similarly, in retrospective studies with non-malformed controls, the magnitude of each of these associations could be estimated by calculating the corresponding odds ratios (*OR*). As illustrated by the three-by-three table in Figure 1b, the ORs for these four associations are as follows:
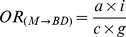
(1)

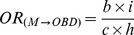
(2)

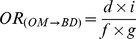
(3)

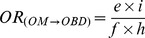
(4)These indicators are known to be sensitive to reporting bias, so different approaches including “malformed” and “only-exposed cases” controls were developed to try to reduce this bias.

Three different case-control designs were used in the present study:

- HEALTHY design: This was the classical case-control design. Cases included those infants with any of the birth defects (alone or in combination with other birth defects). Four non-malformed controls were randomly selected for each case from all healthy newborns registered by ECLAMC in the same hospital and period. These controls showed no difference to total births with respect to maternal age, gravidity, and birth weight (Table S1; supplemental material).
  Subjects were considered exposed if their mothers reported the use of the study medicine during the first trimester of pregnancy (with or without other medications) and were considered non-exposed when their mothers reported no medication use. The magnitude of this association (*OR_HEALTHY_*) was calculated by the same method that was used for *OR_(M→BD)_*, (Figure 1b):
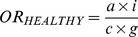
(5)
- SICK design: This was a case-control design in which both the cases and controls were malformed. The cases were defined similarly to those in the HEALTHY design. Four newborns with birth defects other than the case were randomly selected from all malformed newborns registered by ECLAMC in the same hospital and period. The operative definition of exposed versus non-exposed was similar to that for the HEALTHY design. The *OR_SICK_* was calculated as follows (Figure 1b):
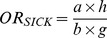
(6)
- OECA (Only-Exposed Cases) design: This approach only included malformed newborns who were prenatally exposed to any type of medicine, so this is actually a subgroup of SICK. Cases and controls were defined similarly to those for the SICK design. Malformed newborns whose mothers reported the use of the study medication were considered exposed subjects, and those whose mothers reported the use of medicines other than the ones studied were included as non-exposed. The OR_OECA_ is represented as follows (Figure 1b):
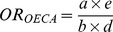
(7)

### Statistical methods and power

Associations between the medicines and birth defects were assessed using Odds ratios (ORs) and Pearson's chi-square test at a level of significance of 1% (P<0.01). Ninety-nine percent confidence intervals (99% CI) were calculated for all birth defects in HEALTHY design. Each birth defect was analysed separately for antiepileptics, insulin, and acetaminophen use during the first trimester of pregnancy.

For the available sample size and a medicine exposure around 1%, the minimum detectable OR is 2.0, with a power of 90% when the sample size is 2000 cases and 60% when sample size is 500 cases. Out of the 31 birth defects analysed in the present study, only two were found in less than 500 cases, and ten were found in more than 2000 cases.

Lin's concordance correlation coefficient (*ρ_c_*) [22] was used as a measure of agreement between the three designs. This method combined measures of precision and accuracy to determine whether the *OR_HELATHY_* and *OR_SICK_* estimates significantly deviated from the line of perfect concordance with the *OR_OECA_* estimates (taken as the baseline). Lin's coefficient increases in value as a function of the nearness of the data's reduced major axis to the line of perfect concordance (a measure of accuracy of data) and of the tightness of the data about its reduced major axis (a measure of precision of data).

Bland-Altman analysis of the limits of agreement [23] was applied to compare the average difference between the designs, together with the variability of the differences and the overall trend. The correlation between the difference and mean (*r_d−m_*) and the 95% limits of agreement (95% CI) were estimated and tested for significance using the Bradley-Blackwood test (F).

Potential reporting/selection biases (in percentages) were calculated according Swan et al. [10] as follows:
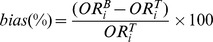
where OR^B^ (“biased” odds ratio) is the observed OR; OR^T^ (“true” odd ratio) is equals to 1, assuming there is not association among acetaminophen use and birth defects; and the letter *i* indicates SICK, OECA, or HEALTHY designs.

For each design, a linear regression model was applied to evaluate possible association between the bias and the proportion of exposed controls to acetaminophen:
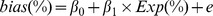
where the β_0_ coefficient is the bias-intercept and the β_1_coefficient evaluate the increment of bias as a function of the proportion of exposed controls (slope of the curve).

All statistical analyses were processed using Stata 12 SE (Stata Corporation, College Station, Texas).

## Results

### Medicine exposure during the first trimester of pregnancy

Out of the 3,939,474 total births that were registered by the ECLAMC, 58,514 newborns had a non-syndromic birth defect, of whom 48,971 had a single birth defect (83.7%), and 9,543 had two or more unrelated major birth defects (16.3%).

Table 1 summarises the frequencies of exposure to medicines during the first trimester of pregnancy among the malformed and non-malformed newborns. Twenty-six percent of the malformed newborns were prenatally exposed to some type of medication, while this percentage was around 19% among non-malformed newborns. Similar relative differences were observed between these groups for exposures to any other medication and for unknown exposures.

**Table 1:** Frequency of exposed to medicines during the first trimester of pregnancy in 58,514 subjects with single or multiple birth defects (excluding minor anomalies and syndromes) and 110,814 non-malformed newborns.

	Malformed newborns		Non-malformed newborns	
	(N = 58,514)		(N = 110,814)	
	N	% (95% CI)	N	% (95% CI)
Total newborns exposed to medicine^1^	15,411	26.3 (26.0–26.7)	20,783	18.8 (18.5–19.0)
Exposed to antiepileptics^2^ (ATC code: N03A)	294	0.50 (0.45–0.56)	254	0.23 (0.20–0.26)
Exposed to insulin^2^ (ATC code: A10A)	228	0.39 (0.34–0.44)	161	0.15 (0.12–0.17)
Exposed to acetaminophen^2^ (ATC code: N02BE01)	1,788	3.06 (2.92–3.20)	2,730	2.46 (2.37–2.56)
Exposed to any other medicine	11,286	19.3 (19.0–19.6)	14,897	13.4 (13.2–13.6)
Unknown exposure	4,215	7.20 (7.00–7.42)	5,285	4.77 (4.64–4.89)
Total Non-exposed newborns^1^	43,103	73.7 (73.3–74.0)	90,031	81.3 (81.0–81.5)

References: (1): Prenatal medication exposure during the first trimester of pregnancy according to maternal report; (2): Exposed to the study medication, alone or in combination with other medications; (ATC code) Anatomical Therapeutic Chemical classification system.

The frequencies of exposure to antiepileptics and insulin for malformed newborns were more than twice those observed for non-malformed babies. A minor difference between these groups was observed in the percentage of exposure to acetaminophen (Table 1). Considering only the total exposed subjects, 1.91% (294/15411), 1.48% (228/15411), and 11.60% (1788/15411) of these infants were prenatally exposed to antiepileptics, insulin, and acetaminophen, respectively.

Table 2 summarises the rate (per 10,000 births) of 30 birth defects and the frequency of medicine exposures during the first trimester of pregnancy registered by the ECLAMC in the study period.

**Table 2:** Number of cases; rate per 10,000 births; and frequencies of exposed to antiepileptics, insulin, and acetaminophen registered by the ECLAMC during 1967–2008.

			Prenatal Medication Exposure^1^							
	Cases		Antiepileptics		Insulin		Acetaminophen		Others	
Birth Defects	N	Rate^2^	N	%	N	%	N	%	N	%
Ambiguous genitalia/Intersexual organs, ambiguous	592	1.5	6	1.0	4	0.7	18	3.0	146	24.7
Anencephaly	1,262	3.2	6	0.5	4	0.3	27	2.1	291	23.1
Anophthalmia	527	1.3	2	0.4	3	0.6	17	3.2	126	23.9
Anorectal atresia/stenosis	1,509	3.8	6	0.4	3	0.2	47	3.1	307	20.3
Atrial septal defect	560	1.4	6	1.1	7	1.3	34	6.1	107	19.1
Axial skeleton malformation	615	1.6	4	0.7	14	2.3	12	2.0	150	24.4
Cardiac left ventricle obstructive defect	548	1.4	1	0.2	9	1.6	20	3.7	115	21.0
Cardiac outflow tract defect	496	1.3	3	0.6	7	1.4	14	2.8	96	19.4
Cardiac right ventricle obstructive defects	615	1.6	3	0.5	2	0.3	27	4.4	119	19.4
Cleft lip with or without paIate	4,267	10.8	32	0.8	11	0.3	115	2.7	838	19.6
Cleft paIate (included Pierre Robin)	1,439	3.7	13	0.9	6	0.4	49	3.4	295	20.5
Cystic kidney	918	2.3	8	0.9	6	0.7	49	5.3	193	21.0
Encephalocele	720	1.8	4	0.6	4	0.6	27	3.8	172	23.9
Gastroschisis	935	9.5	1	0.1	1	0.1	31	3.3	160	17.1
Hip dislocation	5,432	13.8	15	0.3	9	0.2	116	2.1	1061	19.5
Hydrocephaly	3,072	7.8	13	0.4	8	0.3	125	4.1	649	21.1
Hydronephrosis - Ureter stenosis/atresia	2,025	5.1	15	0.7	6	0.3	107	5.3	424	20.9
Hypospadias	3,390	8.6	22	0.7	11	0.3	122	3.6	701	20.7
Intestinal atresia/stenosis	928	2.4	7	0.8	6	0.7	37	4.0	191	20.6
Levo transposition of great arteries	318	0.8	0	0.0	6	1.9	11	3.5	58	18.2
Limb reduction defect	2362	6.0	8	0.3	7	0.3	65	2.8	583	24.7
Microcephaly	1,096	2.8	10	0.9	10	0.9	23	2.1	207	18.9
Multiple malposition/contractures	894	2.3	5	0.6	1	0.1	20	2.2	207	23.2
Oesophageal atresia/stenosis	1,021	2.6	1	0.1	3	0.3	28	2.7	216	21.2
Omphalocele	800	8.1	5	0.6	4	0.5	26	3.3	194	24.3
Patent Ductus Arteriosus	375	1.0	2	0.5	5	1.3	15	4.0	72	19.2
Severe ear malformation	1,838	4.7	3	0.2	13	0.7	46	2.5	362	19.7
Spina bifida	3,058	7.8	29	1.0	5	0.2	94	3.1	625	20.4
Unilateral/Bilateral kidney a/dysgenesis	524	1.3	2	0.4	8	1.5	20	3.8	118	22.5
Ventricular septal defect	2,216	5.6	9	0.4	22	1.0	116	5.2	372	16.8

References: (1): Prenatal medication exposure during the first trimester of pregnancy according to maternal report; (2) Rate per 10,000 live births.

### Concordance among the three case-control approaches

When considering concordance, no significant correlation between the HEALTHY and OECA designs were observed for antiepileptics (*ρ_c_* = 0.07; 95%CI: 0.02–0.11), insulin (*ρ_c_* = 0.06; 95%CI: 0.02–0.10), and acetaminophen (*ρ_c_* = 0.02; 95%CI: 0.01–0.04). The average difference between these designs (*OR_HEALTHY_*−*OR_OECA_*) was 6.1 (95%CI: −1.9–14.0) for antiepileptics, 11.5 (95%CI: −11.8–34.7) for insulin, and 3.0 (95%CI: 0.98–5.06) for acetaminophen. The overestimations observed for HEALTHY design were increased as higher odds ratio values were given, with high and statistically significant correlations between the difference and mean (*r_d−m_*) for antiepileptics (*r_d−m_* = 0.98; F = 863.3; P<0.001), insulin (*r_d−m_* = 0.99; F = 976.9; P<0.001), and acetaminophen (*r_d−m_* = 0.95; F = 1474.9; P<0.001).

In contrast, significant concordance correlations were obtained between the SICK and OECA methods for antiepileptics (*ρ_c_* = 0.95; 95%CI: 0.92–0.98), insulin (*ρ_c_* = 0.98; 95%CI: 0.97–0.99), and acetaminophen (*ρ_c_* = 0.68; 95%CI: 0.51–0.85). There were no significant differences in the average values between *OR_SICK_* and *OR_OECA_* for antiepileptics (0.13; 95%CI: −0.21–0.47), insulin (0.15; 95%CI: −0.25–0.56), and acetaminophen (0.11; 95%CI: −0.17–0.39).

### Significant associations identified by SICK, OECA, and HEALTHY designs

**Figure 2:**
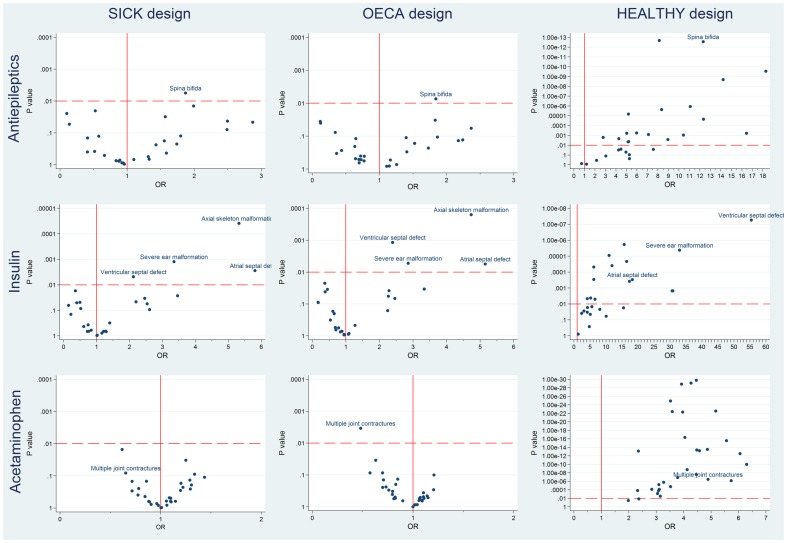
Associations between medications and birth defects for SICK, OECA and HEALTHY designs. The smile plots summarize the associations between each birth defect and exposure to antiepileptics, insulin, and acetaminophen for the three case-control designs. In each smile plot, P-values are plotted on the y-axis on a reverse log scale against the estimated odds ratios on the x-axis. So, the higher the P-values up the y-axis the more significant they are. The vertical full red line represents no association between medication exposure and birth defect (OR equals to 1), and the horizontal dashed red line represents the cut-off P value (0.01).

The associations between each birth defect and exposure to antiepileptics, insulin, and acetaminophen estimated by the three case-control designs are presented as smile plots in Figure 2. In each smile plot, P values are plotted on the y-axis on a reverse log scale against the estimated odds ratios on the x-axis. Therefore, statistically significant positive associations are plotted in the upper right quadrant of each smile plot.

A significant association between antiepileptics exposure and Spina bífida (Q05) was identified by SICK and OECA approaches. While eighteen significant associations (including Spina bifida) with ORs ranging between 2.8 and 18.3 were observed using HEALTHY design (see Figure 2, row 1). Full details of odds ratios, P values and 99% CI estimates for the association between the antiepileptic medications and each birth defect by the three approaches are shown in Table S2 (supplemental material).

Atrial septal defect (Q21.1), axial skeletal malformations (Q67.5; Q76.0–Q76.8), severe ear malformation (Q16.0 and Q17.2), and ventricular septal defect (Q21.0) showed a strong association with insulin exposure using SICK and OECA approaches. Fifteen significant associations (including the four birth defect groups described above) with ORs ranging between 4.2 and 74.6 were identified by HEALTHY design (Figure 2, row 2). All odds ratios, P values, and 99% CI for insulin and each birth defect are presented in Table S3 (supplemental material).

There were no significant associations between the birth defects and acetaminophen (paracetamol) using the SICK and OECA designs, with the unique exception of a negative association (OR<1) with multiple joint contractures (Q74.3) using OECA approach. On the other hand, twenty-nine significant associations with ORs ranging between 2.3 and 6.3 were observed using HEALTHY design (Figure 2, row 3). Details of all odds ratios, P values, and 99% CI for acetaminophen exposure are shown in Table S4 (supplemental material).

Table 3 summarizes the statistically significant results obtained using SICK or OECA approaches. The estimated OR and P values are shown for SICK and OECA designs, while OR and 99%CI are shown for the four measures of association calculated from non-malformed controls.

**Table 3:** Statistically significant results obtained using SICK or OECA approaches.

		Malformed Controls				Non-malformed Controls				
		SICK		OECA		HEALTHY (M→BD)^1^		(M→OBD)^2^	(OM→BD)^3^	(OM→OBD)^4^
Birth Defect	Exposure^5^	OR	P value	OR	P value	OR (99% CI)	P value	OR (99% CI)	OR (99% CI)	OR (99% CI)
Spina bífida	ANTI	1.8	0.006	1.9	0.003	12.3 (5.1–30.0)	<0.00001	6.6 (2.9–14.9)	4.6 (4.0–5.2)	4.5 (4.0–5.0)
Severe ear malformation	INSU	3.4	0.001	2.9	0.005	33.0 (4.6–234.6)	<0.00001	9.9 (1.4–68.2)	5.0 (4.2–6.0)	4.3 (3.7–4.9)
Ventricular septal defect	INSU	2.1	0.005	2.4	0.001	55.5 (8.2–373.3)	<0.00001	26.2 (4.0–169.7)	3.9 (3.3–4.6)	4.4 (3.9–5.0)
Atrial septal defect	INSU	5.8	0.003	5.2	0.006	18.3 (2.3–145.3)	<0.001	3.2 (0.4–27.3)	4.4 (3.2–6.0)	3.9 (3.0–4.9)
Axial skeletal malformations	INSU	5.3	<0.0001	4.7	0.002	74.6 (5.1–689.8)	<0.00001	14 (3.2–96.7)	4.8 (3.6–6.5)	4.3 (3.4–5.4)
Multiple joint contractures	ACET	0.7	0.082	0.5	0.003	4.9 (2.2–10.9)	<0.00001	7.5 (4.1–13.6)	5.9 (4.5–7.6)	4.3 (3.5–5.3)

References: (1): (M→BD) is the risk that the study medication (“M”) causes the birth defect studied (“BD”); (2): (M→OBD) is the risk that the study medication (“M”) produces congenital anomalies other than the birth defect under study (“OBD”); (3): (OM→BD) is the risk that other medicines (“OM”) produce the birth defect studied (“BD”); (4): (OM→OBD) is the risk that other medications (“OM”) cause other birth defects (“OBD”); (5): Exposure: (ANTI) antiepileptic medications, (INSU) insulin, (ACET) acetaminophen.

### Potential bias in each approach

**Figure 3:**
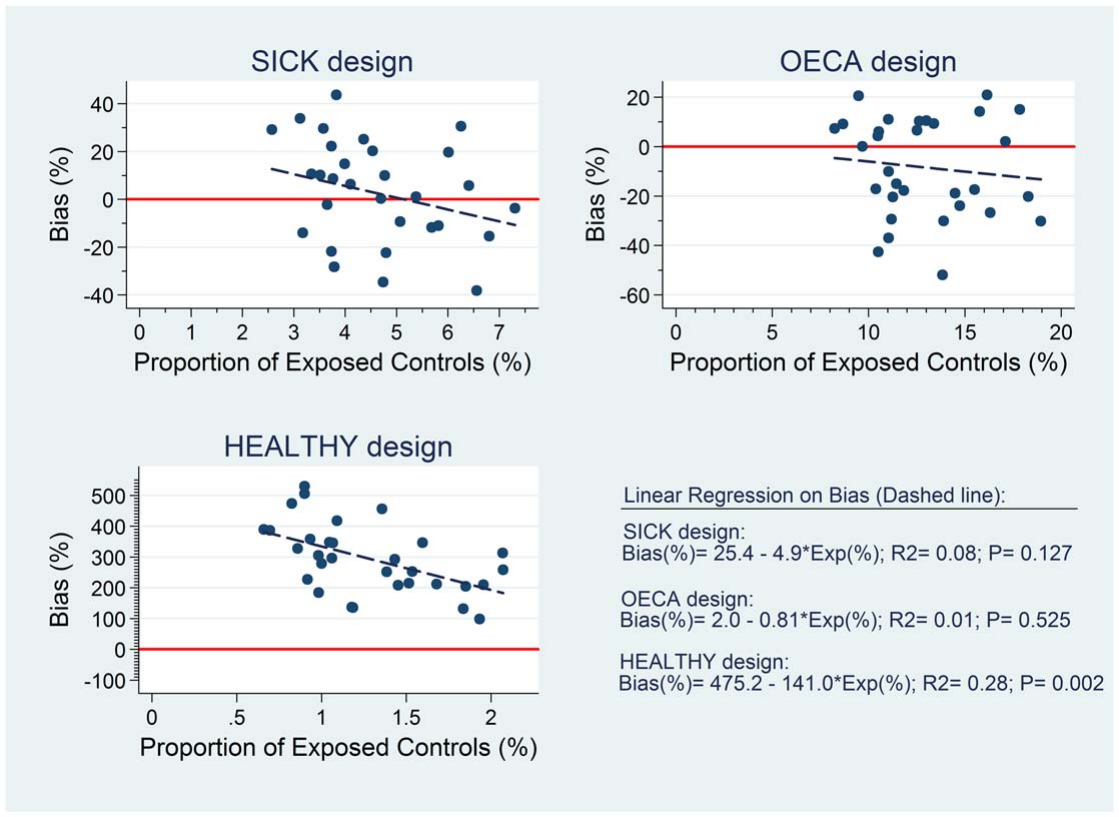
Bias in SICK, OECA and HEALTHY designs. The potential bias (%) is shown as a function of the proportion of exposed controls to acetaminophen (%) in the 31 birth defect groups and for the three evaluated designs. The linear function is defined as follows: Bias(%) = β_0_+β_1_*Exp(%); R-squared (R2) and the overall P value for the regression model are shown for each design.

Figure 3 shows bias as a function of the proportion of exposed controls to acetaminophen for 31 birth defect groups in the SICK, OECA, and HEALTHY designs.

The average proportions of exposure to acetaminophen in the 31 control groups (± SD) were 4.6% (±1.2); 12.9% (±2.9); and 1.3% (±0.4) for SICK, OECA, and HEALTHY design, respectively. While the mean biases (± SD) were 2.6% (±21.7) for SICK; −8.4% (±20.0) for OECA; and 293.4% (±112.1) for HEALTHY design.

No significant associations between the proportion of exposed controls and bias were detected for SICK (β_1_ coeff. = −4.93; P = 0.127), and OECA (β_1_ coeff. = −0.81; P = 0.526) approaches, with overall R-squared of 0.08 (F = 2.46; P = 0.127) and 0.01 (F = 0.41; P = 0.525), respectively. On the other hand, a significant decrease in bias with the increasing proportion of exposed controls was observed for HEALTHY design (β_1_ coeff. = −141.0; P = 0.002), with an overall R-squared of 0.28 (F = 11.3; P = 0.002),

In addition we have calculated the associations between acetaminophen and each of 31 birth defects by the four odds ratios that use non-malformed controls (see Figure 1 and formules 1 to 4). The average odds ratio (± SD) between acetaminophen and the birth defects studied (M→BD) was 3.9 (±1.1). Between acetaminophen and congential anomalies other than the birth defect under study (M→OBD) was 3.9 (±0.9). Furthermore, the average odds ratio observed for other medications and the birth defect studied (OM→BD) was 4.9 (±0.8), and for other medications and other birth defects (OM→OBD) was 4.4 (±0.3).

## Discussion

This study evaluates the scope and limitations of three different approaches used to assess the teratogenic risk of prenatal exposure to medications, applying these methods to the data from ECLAMC's birth-defect surveillance programme. For this purpose, the association between the use of three well-known medicines during the first three months of pregnancy and the risk for 31 types of birth defects were assessed by means of case-control methodology using three types of controls: non-malformed newborns (“HEALTHY” design), malformed newborns (“SICK” design), and malformed newborns exposed to certain medications (“OECA” design).

### Methodological considerations: measures of the association for the HEALTHY, SICK, and OECA designs

The interpretation of the measures of association obtained with the different methodological approaches using non-malformed, malformed, or exposed malformed newborns as the control groups in the research on medication teratogenicity has been extensively discussed [11]–[13], [15], [16], [24]. Although some of the formulas displayed in this work (from No. 1 to No. 14) are certainly basic, we summarised this discussion and applied these basic concepts to real data from a birth defects surveillance programme in order to better understand the relationships among these measures.

Beyond the control group used, these risk estimates from case-control studies of birth defects are vulnerable to both reporting bias and selection bias ([10], [18], [25]. Although effects of selection and reporting bias on the odds ratio are expected to act in opposite directions and it could be difficult to predict in each particular design which are of greater magnitude, both are shown to be algebraically equivalent [10]. Thus, both sources of bias could be combined through a unique term and any of the types of association (Figure 1) estimated by each *OR* calculated from the formulas *(1)* to *(7)* could be expressed as follows:
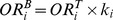
(8)where the observed (“biased”) odds ratio (OR^B^) depends on the true value (OR^T^) and *k* that indicates the effect of bias on the estimated measures of association for each particular approach (*i*).

If cases and non-malformed controls have differential missclasification bias (reporting bias), then *k*_i_>1 and the *OR^B^* will over-estimate the true *OR^T^*. If, however, malformed controls are used and at least some of the malformations in the control group were positively associated with the study medication, this introduces a selection bias so-called teratogenicity non-specific bias [18], then *k*_i_<1 and the *OR^T^* will be under-estimated. Another possibility is that cases and non-malformed controls have poor recall but with non-differential misclassification bias of exposure; or that the use of malformed controls could be to balance out the selective recall by parents of cases, then *k*_i_≈1 and the effect of bias could be considered negligible.

It is important to note that the *k*_i_ term is a function of the ratio of the observed odds of exposure to the true odds of exposure in cases to that in controls. Therefore, it is equivalent to the “selection odds ratio” describe by Kleinbaum [26] and to the inverse of “gamma” (γ) defined by Swan et al. [10].

#### HEALTHY design

This is the classic retrospective case-control design (Figure 1). Considering the formulas *(1)*, *(5)*, and *(8)*, *OR_HEALTHY_* can be expressed as follows:
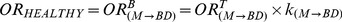
(9)Therefore, this design is unable to reduce the bias (*k_(M→BD)_*) or quantify the magnitude of bias on the estimated *OR_HEALTHY_*, unless the measurement of prenatal exposure has been collected before the birth-defect diagnosis (a prospective cohort design as a “gold standard”).

Our results showed high-sensibility and low-specificity to detect significant associations between each of the three medications and each birth defect using HEALTHY approach. Furthermore, the overestimations that were observed using this design were increased with higher odds ratios. Thus, we might assume that most of these associations are false positive results caused by differential misclassification bias (maternal recall bias or ascertainment bias by the interviewer).

We have shown also that, at least in the case of acetaminophen, this bias effect inflates in average near to 300 percent the odds ratio and that the bias decrease with the increase of proportion of exposed controls. Therefore, if this bias acts in a similar way for different exposures, we expect a higher bias effect for rare medications. This interpretation is in agreement with our observations for antiepileptics and insulin, which have showed very low proportion of exposed and high odds ratios estimated by HEALTHY design for the majority of evaluated birth defects.

The main advantage of using non-malformed controls in retrospective studies of potential teratogens is the possibility of estimating the “true” population odds ratio, but as it is shown in this and previous papers [10], [15], the effects of bias on the observed odds ratio could be considerable and difficult to quantify. One possible approach to quantify this bias when non-malformed controls are used, is to calculate the four associations that has been proposed in this work (formules 1 to 4), under the hypothesis that reporting bias should affect in a similar way the four odds ratios. In this paper, acetaminophen exposure showed average odds ratios around 4 for these associations, showing the lack of specificity of the association between acetaminophen and the study birth defect (M→BD), acetaminophen and other birth defects (M→OBD), of other medications and the birth defect studied (OM→BD), and other medications with other birth defects (OM→OBD). We may regard this value as a rough measure of the magnitude of the overestimation due to reporting bias in this particular study and to use it to adjust the observed measure of association between the study medication and the birth defect studied (M→BD).

Our results are in agreement with the estimate of reporting bias for the retrospective ascertainment of exposure reported by Bar-Oz et al. [15]. These authors investigated the recall bias for itraconazole exposure at least during the first trimester of pregnancy using pharmaceutical-industry data by comparing two cohorts, retrospective and prospective. The authors showed that the chances of the occurrence of a major birth defect after first-trimester exposure to itraconazole were four times higher when the woman or her physician filed the report during the postpartum period than when women were followed up prospectively. As expected, the authors showed that women whose children have major birth defects, or their physicians, are more likely to report the “exposure” than those with healthy newborns.

#### SICK design

This approach uses malformed newborns as the control group. Based on equations *(1)*, *(2)*, *(6)*, and *(8)*, OR_SICK_ can be expressed as follows:
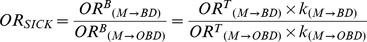
(10)If minor birth defects and well-known associations were excluded from the analysis, and the biases were therefore dependent on the type of medication but to a lesser extent on the birth defect studied, then *k_(M→BD)_≈k_(M→OBD)_*, and consequently:
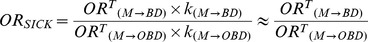
(11)Therefore, the *OR_SICK_* is a measure of the relationship between the risk of the study medication causing the birth defect studied (M→BD) and the risk of the study medication producing congenital anomalies other than the birth defect under study (M→OBD). This *OR_SICK_* is then a measure of the teratogenic specificity of the medication, as previously reported [16]. If the medicine under study is associated with other birth defects, this introduces a type of selection bias known as teratogenicity non-specific bias [18], and then the SICK approach under-reports the true odds ratio. However, if the medications under study has a specific teratogenic effect, then *OR^T^_(M→OBD)_*≈1 and *OR_SICK_* will be a good approximation of the true odds ratio of interest (*OR^T^_(M→BD)_*).

In the present work, acetaminophen exposure showed no significant associations using SICK approach with average odds ratios around 1.0. Moreover, the observed average bias could be considered negligible (2.6%) with no relationship with the frequency of exposure. Thus, unlike *OR_HEALTHY_*, this approach affords the opportunity, under certain assumptions, to reduce the effect of reporting bias on the measure of association. Although it is important to reiterate that *OR_SICK_* is not a direct estimate of the true population odds ratio unless there is no association between the medications and other birth defects.

#### OECA design

This methodology uses malformed newborns exposed to certain medications as the control group. We can develop the previous formulas *(1)*, *(2)*, *(3)*, *(4)*, *(7)*, and *(8)*, and OR_OECA_ can be expressed as follows:
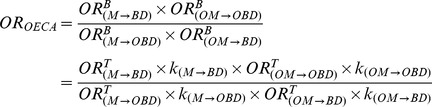
(12)Similar to the SICK design, if the bias is mainly dependent on the kind of medication, then *k_(M→BD)_≈k_(M→OBD)_* and *k_(OM→BD)_≈k_(OM→OBD)_*. On the other hand, if the bias is mainly dependent on the type of birth defect, then *k_(M→BD)_≈k_(OM→BD)_* and *k_(M→OBD)_≈k_(OM→OBD)_*. Under either of these two scenarios, the measure of association can be expressed as follows:
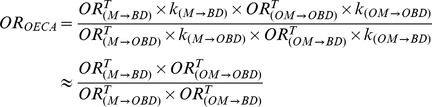
(13)Thus, as previously reported [24], the magnitude of this association depends on a complex relationship between the risk of the study medication to cause the birth defect studied (M→BD), the risk of other medications to cause other birth defects (OM→OBD), the risk of the study medication to produce congenital anomalies other than the birth defect under study (M→OBD), and the risk of other medicines to produce the birth defect studied (OM→BD).

Despite this complex relationship, if the study medication has a specific teratogenic effect (*OR^T^_(M→BD)_>1* and *OR^T^_(M→OBD)_*≈1), and well-known associations are excluded from the analysis (*OR^T^_(OM→OBD)_*≈1), then *OR_OECA_* will be almost equal to the quotient between *OR^T^_(M→BD)_* and *OR^T^_(OM→BD)_*. Therefore, the *OR_OECA_* could be a measure of the degree of aetiological specificity of the defect under study. Then, if the study birth defect were related to other medications, the OECA approach would under-estimate the odds ratio of interest (*OR^T^_(M→BD)_*). However, when the study medication is a major cause of the birth defect under study (*OR^T^_(M→BD)_>1* and *OR^T^_(OM→BD)_*≈1), then *OR_OECA_* will be a good approximation of the true population odds ratio.

In the present study, acetaminophen exposure showed no significant associations using OECA approach and the observed average odds ratio slightly under-estimated the expected value of 1.0. Thus, as for the SICK approach and under certain assumptions, the OECA design could reduce the effect of bias on the measurement of association. Nevertheless, it cannot be considered a direct estimate of the true population odds ratio except under certain conditions as described above.

### SICK vs. OECA designs

Under certain assumptions described in the formulas *(11)* and *(13)*, the relation between *OR_SICK_* and *OR_OECA_* can be expressed as follows:
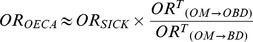
(14)From this relation, and taking into account our results, some general considerations can be outlined:

If the observed odds ratios for the SICK and OECA designs are similar, then it can be inferred that the odds ratio between other medications and other birth defects is equivalent to the odds ratio between other medications and the birth defect under consideration (*OR^T^_(OM→OBD)_*≈*OR^T^_(OM→BD)_*). This was observed in our results, where good concordance correlations were found between SICK and OECA designs for atiepileptics, insulin and acetaminophen. Among the significant associations found, this was mainly evident in the case of antiepileptic and spina bifida.

If *OR_OECA_*>*OR_SICK_*, then it can be concluded that other medication are associated with other birth defects to a greater extent than with the birth defect under study (*OR^T^_(OM→OBD)_*>*OR^T^_(OM→BD)_*). This was observed, for example, for insulin and ventricular septal defects although with small differences in absolute values.

On the other hand, if *OR_OECA_*<*OR_SICK_* we would expect the opposite relationship (*OR^T^_(OM→OBD)_*<*OR^T^_(OM→BD)_*). While only minor differences were observed among the significant associations found, this was the case for, e.g. insulin and severe ear malformation, atrial septal defect, and axial skeletal malformations; and for acetaminophen and multiple joint contractures.

If other medication are associated with other birth defects in a different extent than with the birth defect under study (*OR^T^_(OM→OBD)_*≠*OR^T^_(OM→BD)_*), then only OECA is affected while the SICK is not.

If the study medication is associated with other birth defects apart from the one studied (*OR^T^_(M→OBD)_*>1), then both SICK and OECA will under-estimate the true population odds ratios of interest (*OR^T^_(M→BD)_*).

In view of these considerations and the results discussed in the present work, it is important to exclude all known associations between medications and birth defects from the control groups before obtaining odds ratios values using SICK and OECA designs.

### Findings and comparison of results

#### Antiepileptics

The three approaches identified the recognised association between anti-epileptic exposure during pregnancy and spina bifida. When we used HEALTHY design the odds ratio for spina bifida was twelve times higher than for non-exposed mothers. A cohort study in Finland [27] found a relative risk for spina bifida of 11.3 (95%CI: 2.3–108), which is similar to that from our findings, despite the low accuracy of the estimator. Other recent studies using malformed controls found significant associations between spina bifida and monotherapy with valproic acid [14], [28] and with carbamazepine [29]. Furthermore, using “exposed case-only” (like our OECA approach) and data from twelve registries of congenital birth defects (including ECLAMC), Lisi et al. [11] found significant associations between spina bifida and fatty acid (mainly valproic acid); carboxamide, and other antiepileptic medications.

In the present paper, spina bifida was the only birth defect significantly associated with antiepileptic medications using both SICK and OECA approaches. In addition to spina bifida, previous studies found significant associations between antiepileptics exposure and cardiac defects, cleft lip with or without cleft palate, hypospadias, anomalies of brain, anomalies of circulatory system, limb reduction defects, and hypertelorism [14]; and between antiepileptics exposure and hypospadias, cleft lip with or without cleft palate, polydactyly, cardiac outflow tract defects, cleft palate, limb deficiency, atrial septal defect, and craniosynostosis using OECA design [11].

#### Insulin

Insulin was used as an indicator of clinically significant diabetes, a well-known teratogen that can produce different types of birth defects (see review by Stothard et al. [30]). Severe ear malformation, ventricular septal defect, atrial septal defect, and axial skeletal malformations were significantly associated with insulin by SICK, OECA and HEALTHY designs in our work. With the exception of the first one, all of these associations were in agreement with Lisi et al. [11], who only used the OECA design. Unlike previous reports [11], [30], [31], no relevant associations were detected in our study for cardiac defects, kidney a/dysgenesis, patent ductus arteriosous, holoprosencephaly, choanal atresia, and levo transposition of great arteries.

#### Acetaminophen

For acetaminophen exposure, no significant associations were detected using the SICK design, while an unexpected negative association was found with multiple joint contractures using the OECA approach. The higher odds ratios observed for acetaminophen and other birth defects (*OR_(M→OBD)_*), and for other medications and multiple joint contractures (*OR_(OM→BD)_*) in relation to the two odds ratios in the denominator, could explain this last finding.

Moreover, using HEALTHY design, twenty-nine birth defects were significantly associated with acetaminophen exposure. These results disagree with a recent study conducted by the NBDPS (National Births Defects Prevention Study, USA) that showed that single-ingredient acetaminophen use during the first trimester of pregnancy does not appear to increase the risk for major birth defects [32]. Whereas acetaminophen has no proven teratogenic effect [33], the HEALTHY design in our study increased the number of false-positive associations compared to SICK and OECA designs. Interestingly, we also observed that this bias increases with decreasing proportion of exposed controls. Therefore the differences between the NBDPS study [32] and our study using the HEALTHY design could be related to the ascertainment of exposure and a different selection of controls. While NBDPS reported an average frequency of exposure of 46.9% in cases and 45.8% in controls, we observed frequencies of 3.06% and 2.46%, respectively. The NBDPS study assigned the exposure as “single-ingredient acetaminophen” consumption according to maternal medication use and the information of the Slone Epidemiology Center Drug Dictionary, which identifies product-specific ingredients. While we used a seven-digit ATC code (N02BE01) that could be a more specific exposure classification than that used in the NBDPS study, the use of other drugs together with acetaminophen cannot be ruled out in our study. In addition, the NBDPS study selected the controls from population-based registries, while our work used non-malformed controls from a hospital-based registry.

Finally, the partial discrepancies between our results for the three medications evaluated and previous reports could be due to differences in the sample size, differences in reporting the exposure, differences in the definition of the exposure, differences in the use of a specific medication in different countries, and chance or true differences in exposure risks.

### Strengths and pitfalls

An important issue discussed here is the selection of control group as a potential source of bias. In this regard, it is interesting to consider the strengths and pitfalls of the present work under the framework of the three principles of comparability described by Wacholder et al. in their classical series of papers [2]–[4]: (1) the study base principle, (2) the deconfounding principle, and (3) the comparable accuracy principle.

Because the ECLAMC is a hospital-based program the trade-off between principles No. 1 and No. 3 is a reasonable concern, thus selection bias and information bias cannot be completely discarded. But while the selection bias could affect especially the malformed cases due to referral of prenatally diagnosed cases to hospitals serving high-risk pregnancies; we expect that the referral it be independent of the exposure assessment. Furthermore, the non-malformed controls registered by ECLAMC are not the typical “hospital controls”, that is to say, that were hospitalized for some different disease than cases, but they are selected from the total newborns from each hospital that participates in the ECLAMC program. Thus, the controls are also independently selected of the exposure assessment. In the present work, the non-malformed controls were randomly selected from all healthy newborns registered by ECLAMC in the same hospital and period of time (year) as the cases, and they showed no difference to total births with respect to maternal age, gravidity, and birth weight. In this sense, given that more than 95 per cent of births in South America occur in hospitals, it could be expected that these cases and non-malformed controls are representative samples from the same study population (“the study base”). With regards to principle No. 2, it is plausible that confounding structure may be specific for the study base of each hospital and period of time. Because the confounding by a factor is theoretically eliminated by eliminating variability in that factor, we have selected a random sample of controls born in the same hospital and same year as cases (case-control ratio of 1∶4) to try to control most of the underlying confounding structure.

Another potential pitfall could be that the medications were grouped as acetaminophen, antiepileptics (irrespective of the type of medication), and insulin as a proxy for diabetes. However, we believe there is no major limitation because this study attempts to analyse the performance of case-control studies using three types of controls and does not evaluate the biological significance of the medication exposures and birth defects.

The main strengths in this study are the standardised method in the diagnostic procedures for all malformed and healthy newborns included in the study, and the standardised procedure for medications reported using ATC codes. In addition, medicines and birth defects were reviewed and coded centrally.

## Conclusion

Case-control designs using three control types were compared. The approach using non-malformed controls (HEALTHY) showed a high rate of false-positive results presumably caused by differential misclassification bias. We have shown also that, at least in the case of acetaminophen, this bias decreases with the increase of the proportion of exposed controls. The methods using malformed (SICK) or only-exposed cases (OECA) showed a good concordance for antiepileptics, insulin and acetaminophen. Both approaches could yield similar results, depending on the relationship of the other medications with other birth defects (OM→OBD), and the relationship of other medications with the birth defect under study (OM→BD).

SICK and OECA odds ratios cannot be considered a direct estimate of the true population odds ratio except under certain conditions. However, the SICK design could be effective to determine the teratogenic specificity of the medication, whereas the OECA approach could be useful to estimate the aetiological specificity of the defect under study.

In birth defect surveillance programs that have not access to recruit non-malformed controls, the comparison between SICK and OECA designs could provide practical information to generate hypotheses about potential teratogens.

## Supporting Information

Table S1 Maternal age, gravidity and birth weight differences between the study sample of non-malformed controls and the total births in the period 1967–2008.
(DOC)

Table S2 Odds ratios, 99% confidence intervals, and P values of Antiepileptics exposure for birth defects, according to three case-control approaches: HEALTHY, OECA and SICK designs.
(DOC)

Table S3 Odds ratios, 99% confidence intervals, and P values of Insulin exposure (as a proxy of maternal diabetes) for birth defects, according to three case-control approaches: HEALTHY, OECA and SICK designs.
(DOC)

Table S4 Odds ratios, 99% confidence intervals, and P values of Acetaminophen exposure for birth defects, according to three case-control approaches: HEALTHY, OECA and SICK designs.
(DOC)
