# Supplementary material for: Methodological Approaches to Evaluate Teratogenic Risk Using Birth Defect Registries: Advantages and Disadvantages
Source: PLoS One. 2012 Oct 3;7(10):e46626. doi: 10.1371/journal.pone.0046626 (PMC3463517; doi:10.1371/journal.pone.0046626)
Supplement: Table S1 — Maternal age, gravidity and birth weight differences between the study sample of non-malformed controls and the total births in the period 1967–2008. (DOC) [file pone.0046626.s001.doc]

**Table S1**. Maternal age, gravidity and birth weight differences between the study sample of non-malformed controls and the total births in the period 1967 - 2008.

|  |  | Total Births | |  | Controls | | | |  |  | |
| --- | --- | --- | --- | --- | --- | --- | --- | --- | --- | --- | --- |
|  |  | (N=3,939,474) | |  | (N=110,814) | | | |  | Pearson Chi-square | |
| Variables |  | N | (%) |  | Observed | (%) | Expected | O/E |  | *X^2^* | P value |
| Maternal Age (year) |  |  |  |  |  |  |  |  |  |  |  |
| <=19 |  | 709,895 | 18.64 |  | 19,728 | 18.82 | 19,536 | 1.010 |  | 1.37 |  |
| 20-24 |  | 1,146,921 | 30.11 |  | 31,340 | 29.90 | 31,562 | 0.993 |  | -1.25 |  |
| 25-29 |  | 923,477 | 24.24 |  | 25,363 | 24.19 | 25,413 | 0.998 |  | -0.31 |  |
| 30-34 |  | 609,490 | 16.00 |  | 16,701 | 15.93 | 16,772 | 0.996 |  | -0.55 |  |
| 35-39 |  | 321,039 | 8.43 |  | 8,890 | 8.48 | 8,835 | 1.006 |  | 0.59 |  |
| 40-44 |  | 91,477 | 2.40 |  | 2,625 | 2.50 | 2,517 | 1.043 |  | 2.15 |  |
| >=45 |  | 7,086 | 0.19 |  | 183 | 0.17 | 195 | 0.938 |  | -0.86 |  |
| Total |  | 3,809,386 | 100 |  | 104,830 | 100 | - | - |  | 9.56 | 0.144 |
| Gravidity |  |  |  |  |  |  |  |  |  |  |  |
| 1 |  | 1,289,525 | 34.20 |  | 34,984 | 34.14 | 35,048 | 0.998 |  | -0.34 |  |
| 2 |  | 933,876 | 24.77 |  | 25,710 | 25.09 | 25,382 | 1.013 |  | 2.06 |  |
| 3 |  | 616,255 | 16.35 |  | 16,602 | 16.20 | 16,749 | 0.991 |  | -1.14 |  |
| 4 or more |  | 930,420 | 24.68 |  | 25,172 | 24.57 | 25,288 | 0.995 |  | -0.73 |  |
| Total |  | 3,770,077 | 100 |  | 102,468 | 100 | - | - |  | 6.18 | 0.103 |
| Birth Weight (gr.) |  |  |  |  |  |  |  |  |  |  |  |
| <=1000 |  | 23,460 | 0.60 |  | 557 | 0.53 | 625 | 0.891 |  | -2.72 |  |
| 1001-1500 |  | 40,111 | 1.02 |  | 1,114 | 1.06 | 1,068 | 1.043 |  | 1.41 |  |
| 1501-2000 |  | 84,133 | 2.14 |  | 2,222 | 2.12 | 2,240 | 0.992 |  | -0.38 |  |
| 2001-2500 |  | 266,159 | 6.77 |  | 6,965 | 6.65 | 7,087 | 0.983 |  | -1.45 |  |
| 2501-3000 |  | 941,932 | 23.94 |  | 25,054 | 23.92 | 25,082 | 0.999 |  | -0.18 |  |
| 3001-3500 |  | 1,522,078 | 38.69 |  | 40,689 | 38.84 | 40,531 | 1.004 |  | 0.79 |  |
| 3501-4000 |  | 840,407 | 21.36 |  | 22,494 | 21.47 | 22,379 | 1.005 |  | 0.77 |  |
| 4001-4500 |  | 186,479 | 4.74 |  | 4,916 | 4.69 | 4,966 | 0.990 |  | -0.71 |  |
| 4501-5000 |  | 25,607 | 0.65 |  | 652 | 0.62 | 682 | 0.956 |  | -1.15 |  |
| >=5001 |  | 3,481 | 0.09 |  | 89 | 0.09 | 93 | 0.963 |  | -0.42 |  |
| Total |  | 3,933,847 | 100 |  | 104,752 | 100 | - | - |  | 14.86 | 0.095 |

Percentages are calculated from non-missing data. Missing data on: Maternal Age (total births: 130,088; controls: 5,984); Gravidity (total births: 169,397; controls: 8,346); Birth Weight (total births: 5,627; controls: 6,062).
