# Supplementary material for: Methodological Approaches to Evaluate Teratogenic Risk Using Birth Defect Registries: Advantages and Disadvantages
Source: PLoS One. 2012 Oct 3;7(10):e46626. doi: 10.1371/journal.pone.0046626 (PMC3463517; doi:10.1371/journal.pone.0046626)
Supplement: Table S2 — Odds ratios, 99% confidence intervals, and P values of Antiepileptics exposure for birth defects, according to three case-control approaches: HEALTHY, OECA and SICK designs. (DOC) [file pone.0046626.s002.doc]

**Table S2**. Odds ratios, 99% confidence intervals, and P values of Antiepileptics exposure for birth defects, according to three case-control approaches: HEALTHY, OECA and SICK designs.

|  |  | ANTIEPILEPTIC (ATC code: N03A) | | | | | | | | |
| --- | --- | --- | --- | --- | --- | --- | --- | --- | --- | --- |
|  |  | HEALTHY^1^ | | |  | OECA^2^ | |  | SICK^3^ | |
| Birth Defects | ICD-10 code | OR | CI99% | P value | OR | | P value | OR | | P value |
| Ambiguous genitalia | Q55; Q56 | 16.5 | 2.0 - 135.9 | 0.001 | 2.9 | | 0.046 | 2.2 | | 0.130 |
| Anencephaly | Q00 | 5.2 | 1.2 - 23.2 | 0.004 | 1.3 | | 0.558 | 1.1 | | 0.827 |
| Anophthalmia | Q11.1 | 2.2 | 0.2 - 18.8 | 0.359 | 1.0 | | 0.959 | 0.7 | | 0.650 |
| Anorectal atresia / stenosis | Q42 | 5.2 | 1.2 - 23.0 | 0.005 | 0.9 | | 0.774 | 0.8 | | 0.570 |
| Atrial septal defect | Q21.1 | 10.4 | 1.7 - 65.1 | 0.001 | 2.5 | | 0.079 | 2.2 | | 0.138 |
| Axial skeleton malformation | Q67.5; Q76.0; Q76.1; Q76.3; Q76.4; Q76.5; Q76.6; Q76.7; Q76.8; Q76. | 4.3 | 0.8 - 24.2 | 0.031 | 0.8 | | 0.746 | 0.7 | | 0.511 |
| Cleft lip with or without paIate | Q36; Q37 | 8.1 | 3.9 - 17.0 | 0.000 | 1.6 | | 0.031 | 1.4 | | 0.112 |
| Cleft paIate | Q35; Q87.08 (Pierre Robin) | 11.1 | 3.1 - 39.7 | 0.000 | 1.6 | | 0.179 | 1.4 | | 0.301 |
| Cystic kidney | Q61 | 6.0 | 1.6 - 22.8 | 0.001 | 2.5 | | 0.043 | 2.4 | | 0.058 |
| Encephalocele | Q01 | 4.5 | 0.8 - 25.4 | 0.026 | 1.6 | | 0.431 | 1.1 | | 0.813 |
| Facial dysmorphisms | Q10; Q18.4; Q18.5; Q18.6; Q18.7; Q18.8; Q18.9; Q75.2; Q75.3 | 14.2 | 4.5 - 44.6 | 0.000 | 2.0 | | 0.014 | 1.8 | | 0.033 |
| Gastroschisis | Q79.3 | 1.2 | 0.1 - 21.5 | 0.872 | 0.1 | | 0.025 | 0.1 | | 0.036 |
| Hip dislocation | Q65 | 2.8 | 1.2 - 6.4 | 0.002 | 0.5 | | 0.020 | 0.6 | | 0.120 |
| Hydrocephaly | Q03; G91; G94 | 8.4 | 2.6 - 26.7 | 0.000 | 0.9 | | 0.713 | 0.8 | | 0.409 |
| Hydronephrosis; Ureter stenosis/atresia | Q62 | 5.2 | 2.0 - 13.4 | 0.000 | 1.4 | | 0.237 | 1.5 | | 0.166 |
| Hypospadias | Q54 | 18.3 | 5.6 - 60.1 | 0.000 | 1.1 | | 0.690 | 1.2 | | 0.533 |
| Intestinal atresia / stenosis | Q41 | 7.1 | 1.6 - 32.3 | 0.001 | 1.7 | | 0.223 | 1.7 | | 0.232 |
| L ventricle obstructive defect | Q23; Q25.1; Q25.2; Q25.3; Q25.4 | 5.3 | 0.1 - 204.0 | 0.239 | 0.4 | | 0.396 | 0.4 | | 0.336 |
| Levo transposition of great arteries | Q20.5 | - | - | - | - | | - | - | | - |
| Limb reduction defect | Q71; Q72; Q73 | 4.3 | 1.3 - 14.6 | 0.002 | 0.9 | | 0.885 | 0.7 | | 0.406 |
| Microcephaly | Q02 | 12.3 | 2.7 - 57.0 | 0.000 | 1.8 | | 0.125 | 1.9 | | 0.108 |
| Multiple joint contractures | Q74.3 | 9.0 | 1.4 - 59.2 | 0.003 | 0.9 | | 0.873 | 0.7 | | 0.522 |
| Oesophageal atresia / stenosis | Q39 | 0.7 | 0.0 - 11.6 | 0.772 | 0.1 | | 0.054 | 0.1 | | 0.040 |
| Omphalocele | Q79.2 | - | - | - | 1.5 | | 0.464 | 1.0 | | 0.960 |
| Outflow tract defect | Q20.0; Q20.1; Q20.3; Q20.8; Q20.9; Q21.3; Q25.5 | 5.0 | 0.6 - 41.0 | 0.050 | 1.3 | | 0.670 | 1.3 | | 0.734 |
| Patent Ductus Arteriosus | Q25.0 | - | - | - | 2.1 | | 0.397 | 1.8 | | 0.514 |
| R ventricle obstructive defects | Q22.0; Q22.1; Q22.2; Q22.3; Q22.4; Q22.8; Q24.3; Q25.5; Q25.6 | 7.6 | 0.7 - 80.1 | 0.027 | 0.7 | | 0.512 | 0.6 | | 0.484 |
| Severe ear malformation | Q16.0; Q17.2 | 3.0 | 0.5 - 20.1 | 0.127 | 0.4 | | 0.144 | 0.3 | | 0.077 |
| Spina bífida | Q05 | 12.3 | 5.1 - 30.0 | 0.000 | 1.9 | | 0.006 | 1.8 | | 0.007 |
| Unilateral / Bilateral kidney a/dysgenesis | Q60.0; Q60.3; Q60.6 Q60.1; Q60.4 | 5.3 | 0.4 - 70.1 | 0.096 | 0.5 | | 0.381 | 0.4 | | 0.275 |
| Ventricular septal defect | Q21.0 | 5.0 | 1.5 - 17.0 | 0.001 | 0.6 | | 0.127 | 0.6 | | 0.207 |

**Ref.**: (1) Classical case-control design; (2) A case-control design where both cases and controls were malformed; (3) Only-Exposed Cases design, this approach only includes malformed newborns that were prenatally exposed to any type of medicine.
