# Supplementary material for: Methodological Approaches to Evaluate Teratogenic Risk Using Birth Defect Registries: Advantages and Disadvantages
Source: PLoS One. 2012 Oct 3;7(10):e46626. doi: 10.1371/journal.pone.0046626 (PMC3463517; doi:10.1371/journal.pone.0046626)
Supplement: Table S3 — Odds ratios, 99% confidence intervals, and P values of Insulin exposure (as a proxy of maternal diabetes) for birth defects, according to three case-control approaches: HEALTHY, OECA and SICK designs. (DOC) [file pone.0046626.s003.doc]

**Table S3**. Odds ratios, 99% confidence intervals, and P values of Insulin exposure (as a proxy of maternal diabetes) for birth defects, according to three case-control approaches: HEALTHY, OECA and SICK designs.

|  |  | INSULIN (ATC code: A10A) | | | | | | | | |
| --- | --- | --- | --- | --- | --- | --- | --- | --- | --- | --- |
|  |  | HEALTHY^1^ | | |  | OECA^2^ | |  | SICK^3^ | |
| Birth Defects | ICD-10 code | OR | CI99% | P value | OR | | P value | OR | | P value |
| Ambiguous genitalia | Q55; Q56 | - | - | - | 1.3 | | 0.623 | 1.0 | | 0.976 |
| Anencephaly | Q00 | 4.2 | 0.7 - 23.6 | 0.033 | 1.3 | | 0.663 | 1.1 | | 0.894 |
| Anophthalmia | Q11.1 | 8.1 | 0.8 - 85.8 | 0.022 | 1.3 | | 0.694 | 0.9 | | 0.936 |
| Anorectal atresia / stenosis | Q42 | 15.5 | 0.8 - 305.1 | 0.018 | 0.6 | | 0.433 | 0.5 | | 0.322 |
| Atrial septal defect | Q21.1 | 18.3 | 2.3 - 145.3 | 3.0E-04 | 5.8 | | 0.003 | 5.2 | | 0.006 |
| Axial skeleton malformation | Q67.5; Q76.0; Q76.1; Q76.3; Q76.4; Q76.5; Q76.6; Q76.7; Q76.8; Q76. | 74.6 | 5.1 - 689.8 | 6.5E-09 | 5.3 | | 3.9E-05 | 4.7 | | 1.6E-04 |
| Cleft lip with or without paIate | Q36; Q37 | 6.2 | 1.9 - 19.9 | 4.8E-05 | 0.7 | | 0.373 | 0.7 | | 0.202 |
| Cleft paIate | Q35; Q87.08 (Pierre Robin) | 30.7 | 1.9 - 498.9 | 0.002 | 1.2 | | 0.670 | 1.1 | | 0.845 |
| Cystic kidney | Q61 | 5.2 | 1.2 - 23.3 | 0.004 | 1.0 | | 0.976 | 0.9 | | 0.902 |
| Encephalocele | Q01 | 5.6 | 0.9 - 34.9 | 0.015 | 1.2 | | 0.788 | 0.8 | | 0.756 |
| Facial dysmorphisms | Q10; Q18.4; Q18.5; Q18.6; Q18.7; Q18.8; Q18.9; Q75.2; Q75.3 | 15.7 | 3.5 - 69.9 | 1.9E-06 | 1.4 | | 0.315 | 1.3 | | 0.472 |
| Gastroschisis | Q79.3 | 4.8 | 0.1 - 184.4 | 0.268 | 0.2 | | 0.065 | 0.2 | | 0.091 |
| Hip dislocation | Q65 | 6.2 | 1.7 - 22.8 | 2.9E-04 | 0.5 | | 0.049 | 0.6 | | 0.171 |
| Hydrocephaly | Q03; G91; G94 | 2.4 | 0.8 - 7.3 | 0.039 | 0.5 | | 0.085 | 0.5 | | 0.035 |
| Hydronephrosis; Ureter stenosis/atresia | Q62 | 3.1 | 0.8 - 11.8 | 0.028 | 0.4 | | 0.017 | 0.4 | | 0.022 |
| Hypospadias | Q54 | 11.0 | 2.7 - 44.2 | 9.1E-06 | 0.8 | | 0.607 | 0.9 | | 0.718 |
| Intestinal atresia / stenosis | Q41 | - | - |  | 1.7 | | 0.276 | 1.7 | | 0.286 |
| L ventricle obstructive defect | Q23; Q25.1; Q25.2; Q25.3; Q25.4 | 11.9 | 2.5 - 56.5 | 4.0E-05 | 2.5 | | 0.034 | 2.3 | | 0.057 |
| Levo transposition of great arteries | Q20.5 | 30.9 | 1.9 - 505.0 | 0.002 | 3.5 | | 0.027 | 3.3 | | 0.034 |
| Limb reduction defect | Q71; Q72; Q73 | 4.2 | 1.1 - 15.3 | 0.005 | 1.0 | | 0.951 | 0.8 | | 0.566 |
| Microcephaly | Q02 | 16.5 | 3.0 - 90.2 | 2.2E-05 | 2.2 | | 0.047 | 2.3 | | 0.039 |
| Multiple joint contractures | Q74.3 | 1.3 | 0.1 - 24.1 | 0.791 | 0.2 | | 0.145 | 0.2 | | 0.089 |
| Oesophageal atresia / stenosis | Q39 | 5.1 | 0.6 - 42.3 | 0.045 | 0.8 | | 0.668 | 0.7 | | 0.553 |
| Omphalocele | Q79.2 | - | - |  | 1.0 | | 0.963 | 0.7 | | 0.487 |
| Outflow tract defect | Q20.0; Q20.1; Q20.3; Q20.8; Q20.9; Q21.3; Q25.5 | 17.4 | 2.2 - 138.2 | 3.8E-04 | 2.5 | | 0.056 | 2.5 | | 0.068 |
| Patent Ductus Arteriosus | Q25.0 | 6.6 | 1.2 - 37.6 | 0.005 | 2.6 | | 0.095 | 2.2 | | 0.163 |
| R ventricle obstructive defects | Q22.0; Q22.1; Q22.2; Q22.3; Q22.4; Q22.8; Q24.3; Q25.5; Q25.6 | 10.1 | 0.4 - 238.6 | 0.059 | 0.7 | | 0.675 | 0.7 | | 0.652 |
| Severe ear malformation | Q16.0; Q17.2 | 33.0 | 4.6 - 234.6 | 4.2E-06 | 3.4 | | 0.001 | 2.9 | | 0.005 |
| Spina bífida | Q05 | 4.3 | 0.9 - 20.3 | 0.017 | 0.4 | | 0.051 | 0.4 | | 0.042 |
| Unilateral / Bilateral kidney a/dysgenesis | Q60.0; Q60.3; Q60.6 Q60.1; Q60.4 | - | - |  | 3.0 | | 0.019 | 2.7 | | 0.038 |
| Ventricular septal defect | Q21.0 | 55.5 | 8.2 - 373.3 | 5.5E-08 | 2.1 | | 0.005 | 2.4 | | 0.001 |

**Ref.**: (1) Classical case-control design; (2) A case-control design where both cases and controls were malformed; (3) Only-Exposed Cases design, this approach only includes malformed newborns that were prenatally exposed to any type of medicine.
