# Supplementary material for: Methodological Approaches to Evaluate Teratogenic Risk Using Birth Defect Registries: Advantages and Disadvantages
Source: PLoS One. 2012 Oct 3;7(10):e46626. doi: 10.1371/journal.pone.0046626 (PMC3463517; doi:10.1371/journal.pone.0046626)
Supplement: Table S4 — Odds ratios, 99% confidence intervals, and P values of Acetaminophen exposure for birth defects, according to three case-control approaches: HEALTHY, OECA and SICK designs. (DOC) [file pone.0046626.s004.doc]

**Table S4**. Odds ratios, 99% confidence intervals, and P values of Acetaminophen exposure for birth defects, according to three case-control approaches: HEALTHY, OECA and SICK designs.

|  |  | ACETAMINOPHEN (ATC code: N02BE01) | | | | | | | | |
| --- | --- | --- | --- | --- | --- | --- | --- | --- | --- | --- |
|  |  | HEALTHY^1^ | | |  | OECA^2^ | |  | SICK^3^ | |
| Birth Defects | ICD-10 code | OR | CI99% | P value | OR | | P value | OR | | P value |
| Ambiguous genitalia | Q55; Q56 | 3.1 | 1.4 - 6.7 | 1.7E-04 | 1.1 | | 0.821 | 0.8 | | 0.411 |
| Anencephaly | Q00 | 3.3 | 1.7 - 6.2 | 1.8E-06 | 0.8 | | 0.248 | 0.6 | | 0.035 |
| Anophthalmia | Q11.1 | 5.7 | 2.3 - 14.3 | 7.5E-07 | 1.1 | | 0.620 | 0.8 | | 0.498 |
| Anorectal atresia / stenosis | Q42 | 4.6 | 2.7 - 7.7 | 6.8E-14 | 1.3 | | 0.089 | 1.2 | | 0.293 |
| Atrial septal defect | Q21.1 | 4.1 | 2.2 - 7.6 | 1.9E-09 | 1.3 | | 0.192 | 1.2 | | 0.522 |
| Axial skeleton malformation | Q67.5; Q76.0; Q76.1; Q76.3; Q76.4; Q76.5; Q76.6; Q76.7; Q76.8; Q76. | 2.4 | 1.0 - 5.9 | 0.014 | 0.7 | | 0.294 | 0.6 | | 0.087 |
| Cleft lip with or without paIate | Q36; Q37 | 4.3 | 3.1 - 5.9 | 7.8E-30 | 1.0 | | 0.829 | 0.8 | | 0.136 |
| Cleft paIate | Q35; Q87.08 (Pierre Robin) | 4.5 | 2.7 - 7.5 | 4.4E-14 | 1.2 | | 0.228 | 1.1 | | 0.546 |
| Cystic kidney | Q61 | 5.6 | 3.2 - 9.6 | 2.8E-16 | 1.2 | | 0.285 | 1.1 | | 0.457 |
| Encephalocele | Q01 | 6.3 | 3.0 - 13.1 | 1.1E-10 | 1.4 | | 0.111 | 1.0 | | 0.858 |
| Facial dysmorphisms | Q10; Q18.4; Q18.5; Q18.6; Q18.7; Q18.8; Q18.9; Q75.2; Q75.3 | 5.2 | 3.4 - 7.9 | 3.1E-23 | 1.2 | | 0.171 | 1.1 | | 0.487 |
| Gastroschisis | Q79.3 | 2.3 | 1.3 - 4.1 | 1.6E-04 | 0.6 | | 0.015 | 0.7 | | 0.087 |
| Hip dislocation | Q65 | 2.4 | 1.8 - 3.2 | 8.1E-14 | 0.9 | | 0.148 | 1.1 | | 0.513 |
| Hydrocephaly | Q03; G91; G94 | 3.9 | 2.9 - 5.4 | 1.3E-29 | 1.3 | | 0.033 | 1.1 | | 0.363 |
| Hydronephrosis; Ureter stenosis/atresia | Q62 | 3.6 | 2.6 - 5.0 | 4.1E-23 | 1.0 | | 0.734 | 1.0 | | 0.863 |
| Hypospadias | Q54 | 3.5 | 2.6 - 4.8 | 1.3E-25 | 1.0 | | 0.975 | 1.1 | | 0.560 |
| Intestinal atresia / stenosis | Q41 | 6.1 | 3.2 - 11.5 | 3.1E-13 | 1.1 | | 0.620 | 1.1 | | 0.657 |
| L ventricle obstructive defect | Q23; Q25.1; Q25.2; Q25.3; Q25.4 | 3.1 | 1.5 - 6.5 | 7.7E-05 | 0.9 | | 0.621 | 0.8 | | 0.300 |
| Levo transposition of great arteries | Q20.5 | 3.1 | 1.2 - 8.6 | 0.003 | 0.9 | | 0.775 | 0.8 | | 0.557 |
| Limb reduction defect | Q71; Q72; Q73 | 4.0 | 2.6 - 6.2 | 4.8E-17 | 1.1 | | 0.497 | 0.8 | | 0.199 |
| Microcephaly | Q02 | 2.8 | 1.4 - 5.6 | 7.9E-05 | 0.7 | | 0.149 | 0.7 | | 0.143 |
| Multiple joint contractures | Q74.3 | 4.9 | 2.2 - 10.9 | 3.7E-07 | 0.7 | | 0.082 | 0.5 | | 0.003 |
| Oesophageal atresia / stenosis | Q39 | 3.8 | 2.0 - 7.3 | 1.3E-07 | 1.1 | | 0.643 | 1.0 | | 0.997 |
| Omphalocele | Q79.2 | 4.5 | 2.2 - 8.9 | 2.5E-08 | 1.3 | | 0.256 | 0.9 | | 0.652 |
| Outflow tract defect | Q20.0; Q20.1; Q20.3; Q20.8; Q20.9; Q21.3; Q25.5 | 2.0 | 0.9 - 4.5 | 0.033 | 0.8 | | 0.395 | 0.7 | | 0.248 |
| Patent Ductus Arteriosus | Q25.0 | 3.0 | 1.3 - 7.2 | 0.001 | 1.0 | | 0.972 | 0.8 | | 0.537 |
| R ventricle obstructive defects | Q22.0; Q22.1; Q22.2; Q22.3; Q22.4; Q22.8; Q24.3; Q25.5; Q25.6 | 3.1 | 1.6 - 5.9 | 6.1E-06 | 0.8 | | 0.444 | 0.8 | | 0.329 |
| Severe ear malformation | Q16.0; Q17.2 | 4.9 | 2.8 - 8.3 | 3.2E-14 | 1.3 | | 0.137 | 1.1 | | 0.623 |
| Spina bífida | Q05 | 4.0 | 2.8 - 5.7 | 5.2E-23 | 1.1 | | 0.483 | 1.1 | | 0.634 |
| Unilateral / Bilateral kidney a/dysgenesis | Q60.0; Q60.3; Q60.6 Q60.1; Q60.4 | 3.5 | 1.6 - 7.5 | 1.9E-05 | 0.9 | | 0.645 | 0.7 | | 0.240 |
| Ventricular septal defect | Q21.0 | 4.5 | 3.2 - 6.2 | 1.8E-30 | 1.1 | | 0.607 | 1.2 | | 0.101 |

**Ref.**: (1) Classical case-control design; (2) A case-control design where both cases and controls were malformed; (3) Only-Exposed Cases design, this approach only includes malformed newborns that were prenatally exposed to any type of medicine.
